# miRNAs in Insects Infected by Animal and Plant Viruses

**DOI:** 10.3390/v10070354

**Published:** 2018-07-03

**Authors:** Verna Monsanto-Hearne, Karyn N. Johnson

**Affiliations:** School of Biological Sciences, The University of Queensland, Brisbane 4072, Australia; v.hearne@uq.edu.au

**Keywords:** miRNA, insect virus, arbovirus, plant virus, host-virus interaction

## Abstract

Viruses vectored by insects cause severe medical and agricultural burdens. The process of virus infection of insects regulates and is regulated by a complex interplay of biomolecules including the small, non-coding microRNAs (miRNAs). Considered an anomaly upon its discovery only around 25 years ago, miRNAs as a class have challenged the molecular central dogma which essentially typifies RNAs as just intermediaries in the flow of information from DNA to protein. miRNAs are now known to be common modulators or fine-tuners of gene expression. While recent years has seen an increased emphasis on understanding the role of miRNAs in host-virus associations, existing literature on the interaction between insects and their arthropod-borne viruses (arboviruses) is largely restricted to miRNA abundance profiling. Here we analyse the commonalities and contrasts between miRNA abundance profiles with different host-arbovirus combinations and outline a suggested pipeline and criteria for functional analysis of the contribution of miRNAs to the insect vector-virus interaction. Finally, we discuss the potential use of the model organism, *Drosophila melanogaster*, in complementing research on the role of miRNAs in insect vector-virus interaction.

## 1. Introduction

Viruses vectored by arthropods pose severe medical and agricultural, and therefore social and economic burdens. Medically, there are over 100 known human pathogens that are transmitted by insects, mainly dipteran blood-feeders such as mosquitoes [1,2]. Examples of relevant arthropod-borne viruses (arboviruses) are the Zika virus (ZIKV), West Nile virus (WNV), chikungunya virus (CHIKV) and dengue virus DENV [3,4,5]. DENV alone infects up to 390 million people per year [6], leading to around 20,000 deaths [7]. Agriculturally, the majority of plant viruses depend on hemipteran sap-feeders for plant-to-plant transmission [8,9,10]. Some of these plant viruses do not need to replicate in the insect vector. While others, termed circulative, propagative viruses or phytoarboviruses [11], require replication in their vectors as a prerequisite to transmission. Examples of important phytoarboviruses include rice stripe virus, rice black-streaked dwarf virus, rice hoja blanca, and tomato spotted wilt virus (TSWV). TSWV, in particular, which infects thousands of plant species, can cause total loss of crops [12], and is responsible for annual economic losses of over $1 billion in the USA alone [13,14].

Biological transmission of arboviruses and phytoarboviruses occur in an epidemiological cycle involving alternating insect-virus and non-insect-virus associations. Insects acquire animal and plant viruses by feeding on an infected non-insect host and, consequently, ingestion of the virus into the insect gut. In the insect vector, virus replicates in the midgut epithelial cells, spreads into other tissues via the hemocoel and is eventually released into the salivary ducts where the virus exits the insect and is introduced into a new host during feeding (reviewed in References [11,12,15,16]).

Currently, the majority of host-virus interaction literature is focused on the non-insect host [17]. Two key differences exist between insect-virus and non-insect-virus associations during transmission of arboviruses and phytoarboviruses. The first is that while viruses vectored by insects can be highly pathogenic and lethal to animal and plant hosts, the virus usually does not confer a major fitness cost in the insect host. The typical non-pathogenic phenotype exhibited by insects upon arbovirus infection is attributed to insect tolerance [17] and/or the robust arthropod innate immune response controlling viral infections [18]. However, the full inventory of mechanistic interactions and genomic factors that enable the insects to control viral pathogenicity is not yet complete. Understanding the molecular bases for insect-virus interactions in comparison to non-insect-virus interaction would clarify the determinants that enable the persistent phenotype observed in insects infected by the viruses they vector. The second difference between insect-virus and non-insect-virus associations is that whereas the animal and plant hosts often serve as short-term high-titre reservoirs of virus for infection of more insect hosts, virus infection of the insect host/vector is usually persistent [11,19]. Thus, arthropods have greater influence on the survival and evolution of arboviruses [11,19]. Understanding virus replication in and transmission by insect hosts could identify pathways which are suitable for intervention. This means that research on insect-virus interactions could facilitate the design of measures for controlling arbovirus and phytoarbovirus infection and transmission.

Virus infection of insects induces an antiviral host response, central to which is the RNA interference (RNAi) (reviewed in [20,21,22,23,24,25,26,27,28,29]). There are at least three identified pathways in this ancient, cell-intrinsic, and broad acting mechanism: Piwi-interacting RNAs (piRNA), small interfering RNAs (siRNA), and microRNAs (miRNA). miRNAs are canonically derived from transcribed miRNA precursors which are encoded in protein coding or non-coding transcription units in the genome by RNA polymerase II. These primary RNA transcripts fold back into dsRNA stem-loop structures and are cleaved sequentially by Drosha in the nucleus and then Dicer in the cytoplasm. One of the strands of the resulting ~22 nt dsRNA then guides the Argonaute to mRNA sequences complementary to the miRNA [30,31,32,33,34,35,36,37,38,39]. Because perfect binding of even just the 2nd to the 8th molecule from 5′ end of miRNA (seed region) may be sufficient for miRNA function, a single miRNA can regulate many genes. Conversely, a single mRNA can include target sites for multiple miRNAs. 

This review focuses on existing and prospective research on miRNAs in arbovirus and phytoarbovirus infection of insects. The role of miRNAs in virus infection of animal and plant hosts is already comprehensively reviewed elsewhere [40,41,42,43,44,45,46,47,48,49,50,51]. The first part of the review systematises current data on miRNA profile changes in insects during arbovirus and phytoarbovirus infection, showing that progress in research on the role of miRNAs in insect-virus infection has been comparatively slow, and literature remains scant [52]. Of the various medically and agriculturally important insect-vectored animal and plant viruses, only a few miRNA profile studies on insect vector-virus interaction are available. Even fewer data exist on the functional role of miRNAs in insect-virus interactions, and these are summarised in the second part of the review. With a view to stimulate further research in this area, a miRNA functional analysis guideline is outlined to provide a suggested pipeline and criteria for determining whether a miRNA-mRNA target association is relevant for the vector-virus interaction. Finally, upon recognition that the scarcity in vector-virus interaction literature is partly an attribute of the onerous nature of performing molecular and genetic studies in insect vectors, the third part of the review considers *Drosophila melanogaster* as a model organism to augment studies on the role of miRNAs in insect-virus interactions.

## 2. miRNA Profile Changes in Insects during Virus Infection

Changes in host miRNA profile are commonly observed upon stimuli effected by virus infection [41,44,48,52,53,54,55,56,57,58,59,60,61,62]. In fact, differential abundance of miRNAs during viral infection is most often used as the primary determinant of which miRNAs possibly have roles in host-virus interaction [52]. There are three main platforms which are used for miRNA profiling: Reverse transcription-quantitative PCR (RT-qPCR), microarray hybridization, and next generation sequencing (NGS). While each one of the three have their own strengths and weaknesses in terms of reproducibility, sensitivity, accuracy, specificity, and concordance of differential expression (reviewed in [63]), NGS is gaining popularity as the choice platform for initial miRNA profiling studies [54]. There are many comparative NGS studies for uninfected versus virus-infected mammals [52], however, only a few studies have been reported for insects infected with arboviruses (outlined in Table 1). The mosquito-DENV is the most-studied vector-virus pair, with samples coming from intra-thoracically inoculated whole insects, orally infected whole insects, midgut from orally infected mosquitoes, and finally cultured mosquito cells. Mosquito-CHIKV is the next most-studied pair, with samples coming from saliva and cells. For ZIKV and WNV infections, samples only come from whole insects and from cultured cells for blue-tongue virus (BTV) infection. Sampling times post-infection greatly vary, ranging from 1 to 14 days post-infection (dpi). Given the diversity of parameters, the existing information on miRNA profile changes is somewhat fragmented, however, some patterns, upon compilation of available data can be inferred. In *Aedes* (details in Table 2),

1. ***Some miRNAs are commonly differentially abundant due to various arbovirus infections.*** Bantam/bantam-3p, for example, is differentially-regulated in whole *Ae. aegypti* infected by DENV [64], whole *Ae. albopictus*-infected by DENV [65], *Ae. aegypti* infected by CHIKV, and whole *Ae. albopictus*-infected by CHIKV [66]. miR-263a and miR-34 are also differentially abundant in 4 different insect-virus pairs [65,66,67]. This suggests that there may be core miRNAs which are regulated during viral infection of insects, regardless of the virus, sample source (whole insect, body part, or cells), and time of harvest. It would be interesting to systematically explore the regulation and function of this set of core miRNAs as they pertain to host-virus interaction.
2. ***The number of differentially regulated miRNAs changes throughout the course of infection.*** In a study looking at the effect of ZIKV infection on *Aedes* at 2, 7, and 14 days post-infection, it was noted that 10 miRNAs are differentially abundant at day 2 post-infection. At 7 dpi, this number is reduced to 7 miRNAs. At the last time-point (14 dpi), there were only 6 differentially abundant miRNAs [67]. In a study looking at the effect of DENV infection on *Aedes* at 2, 4, and 9 dpi, 5 miRNAs were differentially abundant at 2 dpi. This increased to 27 at 9 dpi [64]. It is interesting to note that the lowest number of differentially regulated miRNAs in ZIKV-infected mosquitoes was observed at 14 dpi when ZIKV load was highest, while the highest number of differentially regulated miRNAs were found in 2 dpi samples when ZIKV titres were still very low [67]. Additionally, the highest number of differentially regulated miRNAs in DENV-infected mosquitoes were found at 9 dpi when 50% of the samples have DENV levels that are below plaque titration-detectable amounts. Taken together these studies show that the miRNA profile is very dynamic and highlights that a single time point may not be sufficient to fully understand the impact of virus infection on the miRNA profile. This is consistent with the dynamic gene regulation observed during a virus infection.
3. ***The direction of regulation of a miRNA can change depending on the time post infection.*** Some miRNAs are regulated only at specific time-points, while others are regulated at different time points. Of those which are regulated at different time points, some can take a singular direction, such as miR-2945-5p which is down-regulated in both 4 dpi and 9 dpi in the study looking at the effect of DENV infection on *Ae. aegypti* at 2, 4, and 9 dpi [64]. Other miRNAs can have opposite directions of regulation, depending on time of sampling. For example, in the study looking at the effect of ZIKV infection on *Aedes* at 2, 7, and 14 days post-infection, miR-308-5p was up-regulated at 2 dpi, but down-regulated at 5 dpi [67]. The differential regulation of individual miRNAs along the course of infection could be a reflection of the attack and counter-attack mechanisms occurring between the insect and virus during the progression of infection. Thus, for a complete picture of the interaction, miRNA dynamics over time are important.
4. ***The general direction of miRNA regulation varies depending on tissue sample source.*** In whole mosquito samples infected with ZIKV [67] and DENV [64,65], the majority of miRNAs (75% or more) are down-regulated, while only 25% or less are up-regulated. However, in midgut samples, where the viral replication is most active, around 90% of the analysed miRNAs are up-regulated during DENV infection [68]. Similarly, in saliva samples, which have been shown to contain factors that enhance viral replication in the vertebrate host [69,70,71], around 80% of the miRNAs are up-regulated during CHIKV-infection [66]. Whereas the whole-body samples provide the global net change in miRNA levels, the tissue-specific samples provide the local miRNA profiles. Considering that the level of viral activity and viral titres change from initial ingestion in the foregut to infection of the midgut and then dissemination to secondary tissues including the salivary glands and finally transmission through saliva [72], it would be interesting to examine the regulation of miRNAs in different tissues and correlate the results with the location of viral activity and viral titres. In addition, because only a few cells are actually infected and become sites of viral replication (reviewed in Reference [72]), it would be interesting to examine the regulation of miRNAs in the infected cells and compare with the regulation of miRNAs in the uninfected cells in the same tissue and in the whole organism. miRNA profiling at the cellular level would provide a higher resolution view of miRNA regulation during infection and insights into the heterogeneity of miRNA profile regulation in infected versus uninfected cells.

For phytoarboviruses, only rice black-streaked dwarf virus (RBSDV) infection of leafhopper, *Laodelphax striatellus*, has been examined at the level of miRNAs. Using NGS, identities and expression levels of miRNAs in virus-free and RBSDV samples were compared. Twenty-one miRNAs were found to be differentially abundant—9 were found to increase in numbers upon RBSDV infections, while 12 were found to decrease in copies [77].

## 3. Functional Role of miRNAs in Insect-Virus Interaction

**Figure 1:**
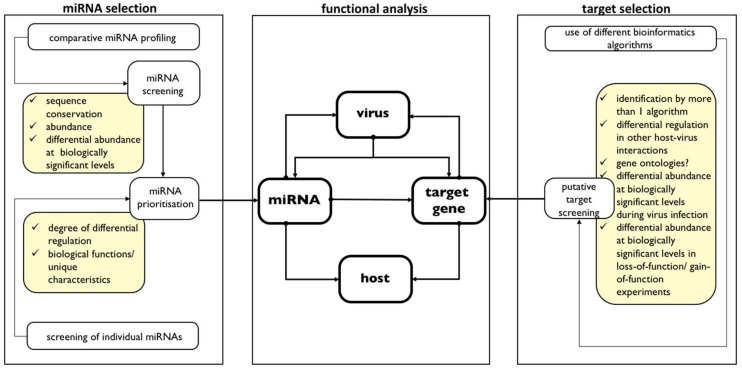
Suggested pipeline and criteria for analysis of the miRNA function in host-virus interaction. Identification of miRNAs with important roles in host-virus interactions typically starts with miRNA profiling. Comparison of uninfected and infected samples commonly identify many miRNAs with differential abundance upon infection. Especially for pilot studies, miRNA screening and miRNA prioritisation using the outlined criteria (in yellow-shaded boxes, left panel) will help streamline and focus research. Identification of targets by bioinformatics also generate many putative mRNA targets. Screening using the outlined criteria (in yellow-shaded boxes, right panel) will as well aid in narrowing down the putative targets. Once miRNA of interest has been identified, and its target confirmed, a closed-loop inter-relationship amongst the (1) miRNA, (2) target gene, (3) virus, and (4) host has to be confirmed (centre panel) to categorically identify the miRNA-target pair as being relevant in the host-virus interaction. Where LOF/GOF mutants exist, identification of relevant miRNAs can start with genetic screens (left panel).

Comparative miRNA profiling is only a starting point in determining the role of miRNAs in the host-virus interaction. Typically, many miRNAs are differentially abundant between uninfected and infected samples. The process of confirming a functional impact of a differentially abundant miRNA involves a large research commitment. Therefore, it is helpful to have criteria for selecting which miRNAs to pursue for further analysis for pilot studies. We suggest two-tier miRNA selection criteria (see Figure 1, left panel) which will first screen for miRNAs which are likely to be functional in the host-virus interaction and then identify which of the miRNAs to prioritise.

The first tier of miRNA selection criteria will identify miRNAs which are most likely to be functional in host-virus interaction using sequence conservation, miRNA abundance, and statistically- and biologically-significant differences between the uninfected and infected samples as bases. The requirement for sequence conservation is on the premise that functional miRNAs are important and, as a consequence are conserved [78]. The sequence homology cut-off score of >70% over the miRNA length can be adopted as this score has been shown to identify most miRNA homologs [79]. The criterion for miRNA abundance is based on studies on miRNA concentration and activity that suggest a requirement for a threshold miRNA level to be reached before any significant effect on host transcripts can occur [47,80,81]. A conservative estimate for the minimum copy number needed for miRNAs to be biologically relevant is around 100 copies per cell [47]. In terms of reads per million (RPM), which is the usual quantification for next-generation sequencing, it has been suggested that it is at levels of above 1000 RPM that 80% of miRNAs are found to regulate their targets [80] and at log_10_-transformed abundances higher than 3.6 (approximately 4000 RPM) that miRNAs are found to repress their targets by more than 50% [82]. This criterion does not imply that less abundant miRNAs are irrelevant as there are studies indicating that low abundant miRNAs can have biological impacts. In addition, in the bigger picture, low miRNA abundance could just mean that the miRNA is less abundant in particular tissues sampled. In pilot studies mainly using whole samples, however, this criterion is very helpful as it would typically identify around 20 miRNAs given that normally only less than 20 miRNAs make up >80% of all miRNAs [83].

The remaining points of the first tier of suggested miRNA selection criteria defines which miRNAs are likely functional specifically in a host-virus interaction. Two key requirements are suggested: Statistically- significant difference and >1.5-fold expression difference between the uninfected and infected samples. Statistically significant difference increases the likelihood that the differential miRNA abundance between the uninfected and infected samples is due to virus infection. Note that statistical analysis also underscores the need for biological replicates. The 1.5-fold expression difference cut-off is based on previous miRNA profiling studies indicating that >1.5-fold difference results to biologically significant effects on cells [63,84,85,86,87,88,89]. It should be noted that screening for miRNAs with >1.5-fold change upon viral infection must be preceded by screening for abundant miRNAs because small fold changes in an abundant miRNA will have a significant effect on target genes, however even a large fold-change for a miRNA that is present in low levels may not lead to measurable cellular changes. For example, if the uninfected sample has 0.1 reads per million (rpm) for the miRNA of interest and the infected sample has 10 rpm, the fold change will be 100×. While this is definitely higher than the subscribed 1.5× cut-off for biological significance, this may not translate to an observable phenotypic trait because the absolute number of miRNAs may not be sufficient for targeting the quantity of mRNAs present in the cell. It is therefore important to apply the abundance cut-off first before applying the differential abundance cut-off for pilot studies.

The use of these suggested criteria should sufficiently narrow down the number of miRNAs such that it becomes realistic to validate the differential abundance of miRNAs using RT-qPCR, the profiling platform that has the overall better score in terms of sensitivity and accuracy in comparison to other technologies [63]. This should be done on at least 3 independent biological replicates, and differential abundance should be statistically significant and at biologically-relevant levels of >1.5-fold for the same reasons stated above. As described above, miRNA profiles are snapshots based on sample source and timing of sampling. Therefore, different miRNA profile changes due to sample source and timing may be observed. In effect, different miRNAs may emerge as being abundant and differentially regulated in various samples. This underscores the value of examining miRNA profiles using different samples at higher resolutions (from organism, to tissue, to cells) and at different time-points. For pilot studies, however, the suggested criteria should direct research towards key miRNAs.

The second tier helps to prioritise which of the miRNAs to pursue. Previous annotation of miRNAs with biological function, degree of differential abundance, differential abundance in different host-virus pair, and unique biological characteristics may be used. In using previous annotation as a criterion, searching curated databases of miRNA-target interactions, such as TarBase [90], miRTarBase [91], miRecords [92], and OncomiRDB [93] are good places to start. This should however be complemented by a thorough search of the current literature as the databases may not necessarily be up-to-date. Looking at previous annotations is useful when identification of miRNAs with unknown functions is not central to the research question. If, however, the identification of miRNAs with unknown functions is important, or if none of the miRNAs have known functions, comparing the degrees of differential abundance may be used. This is an easy way to rank the miRNAs and is on the premise that a large change in miRNA levels may mean either that the miRNA is highly impacted by the virus infection or that the miRNA is highly regulated by the virus or the host.

Unique biological characteristics of miRNAs can also be used as basis for selection. For example, miR-281 was specifically chosen in a study looking at DENV infection of *Ae. albopictus* because of miR-281’s unique characteristic of being highly expressed in both sugar-fed and blood fed female mosquitoes [94]. Another miRNA that was chosen for further research due to its unique biological characteristic is *Ae. aegypti* miR-2940. miR-2940 is induced in mosquitoes infected by *Wolbachia*, an endosymbiont that suppresses viral accumulation [95], and as such was examined for its possible role in DENV inhibition in *Ae. aegypti* [96,97]. Differential abundance in a different host-virus pair can as well be used as a criterion. In the study looking at miRNA role in WNV infection of *Aedes*, miR-2940 was specifically examined [98] because of the miRNA’s known role in restricting DENV [97]. The determination of roles of a miRNA in different host-virus pairs can lead to discovery of miRNAs with broad-range host-virus involvement.

When miRNAs of interest have already been identified, the effect of differential abundance of miRNA on host-virus interaction can be examined. Testing the effect of changes in miRNA levels during virus infection necessitates manipulation of miRNA levels by gain-of-function (GOF) or loss-of-function (LOF) methods [99,100]. In the miRNA functional studies thus far carried out in the context of insect host-arbovirus interaction, chemically synthesized, double-stranded RNAs which mimic mature endogenous miRNAs are typically used for GOF experiments [101,102,103]. Termed miRNA mimics, these synthetic RNA strands artificially simulate increase in miRNA levels by augmenting endogenous miRNA function. Other common strategies for GOF include the use of an expression vector containing mature miRNA, precursor miRNA, or pri-miRNA sequence [99,103]. For miRNA loss-of-function analysis, chemically synthesized, single-stranded RNA molecules designed to specifically bind to and inhibit endogenous miRNAs are commonly used. Termed miRNA inhibitors, these molecules artificially down-regulate miRNA levels by suppressing the function of endogenous miRNAs [104]. Other common strategies for LOF include miRNA sponges, tough decoys, target protectors, miRNA activity sensor, and target sensor [83,105]. To test the effect of changes in miRNA abundance on host-virus interaction, miRNA mimics and inhibitors are typically transfected to cells and biological phenotypes such as cytopathic effects, host survival, and virus titres are then measured. Cells should also be transfected with synthetic double-stranded and single-stranded oligonucleotides with scrambled sequences as controls for mimics and inhibitors, respectively. miRNA mimics and inhibitors have also been successfully introduced to whole organisms per os [106] or through injection into the thorax of mosquitoes [94]. GOF and/or LOF of a miRNA that is involved in host-virus interaction should lead to changes in parameters of host-virus association [101].

Table 3 enumerates the available data on the effects of artificial manipulation of miRNAs on arbovirus infection in an insect host. It also shows that a single miRNA mimic/inhibitor can have different effects on virus accumulation depending on the time post-infection. For example, in the study looking at the effect of miR-12 inhibitor on CHIKV infection at 24, 48, and 72 h post-infection (hpi), it was found that while a decrease in miR-12 results in greater virus density at 24 and 72 hpi, it resulted in lower virus density at 48 hpi [66]. These results may correlate with the observation that some miRNAs are differentially regulated at different time points (see first section of the review). There are no available data showing whether miR-12 levels change throughout the course of infection, but it would be interesting to know the validity of this hypothesis.

Following validation of miRNA differential regulation upon viral infection of host, the next step in miRNA functional analysis is target identification. This is a crucial step in understanding the role of miRNA in the host-virus interaction because miRNA impact is exerted through its targets. While it took 6 years to realise the interaction between the first discovered miRNA-target pair, lin-4-*lin-14* [110,111], we have at our disposal many computational prediction tools that generate a list of putative targets as starting point [102,103,105,112,113]. To narrow down the list of targets to screen, many studies start with putative targets that are identified by more than one algorithm (see Figure 1, right panel). Additionally, many studies use gene homologies and ontologies as criteria for choosing targets to prioritise. In fact, in arbovirus infection of insects, the targets of a number of miRNAs, example miRs-12 [107], 252 [109], 281 [94], 375, and 2940-5p [95,96,97] (shown in Table 3), have been identified using gene ontologies. Finally, there also now exists several experimental techniques and tools for validation of miRNA-target pairing. The readers are directed to several excellent reviews on target identification and validation [102,103,105,112,113].

It is important to note that miRNAs can have many mRNA targets. Therefore, confirmation of a functional interaction between a miRNA known to be involved in virus infection and mRNA, does not necessarily mean the mRNA is involved in the host-virus interaction. The functional impact of the target mRNA in the host-virus interaction needs to be experimentally evaluated. As with assessment of the effect of changes in miRNA levels on host-virus interaction, the assessment of the impact of changes of target levels requires manipulation of the target levels by GOF or LOF. The use of RNAi to knockdown the target gene is the most commonly used technique in insect miRNA functional studies. For example, RNAi was used to deplete miR-2940 target metalloprotease m41 FtsH and show that its depletion restricts WNV replication [98], confirming that the identified target is functional in host-virus interaction. 

Figure 1 centre panel shows how (1) a miRNA, (2) its target, and the determinants of host-virus interaction, specifically (3) virus accumulation and (4) host mortality, are related in a closed-loop manner if the miRNA and the identified target are both functional in host-virus interaction. In this miRNA-target host-virus interaction:

1. Virus infection changes miRNA abundance;
2. Virus infection changes target gene levels;
3. Changes in miRNA affects target levels; changes in target levels during virus infection is because of changes in miRNA abundance levels;
4. Changes in miRNA levels affect pathogenicity determinants in virus (example: replication) and/or in host (example: delay in mortality); because
5. Changes in target levels affect pathogenicity determinants in virus (example: replication) and/or in host (example: delay in mortality).

The use of *in vivo* systems for looking at the regulation of miRNA to the analysis target role in host-virus interaction (with the exception of direct target validation which almost invariably uses cell systems) allow for a spatially and temporally-synchronised data. While *ex vivo* miRNA studies have mainly contributed to the identification of miRNA targets and, as well, to our understanding of functions of miRNAs, it is the *in vivo* studies that truly show whether a miRNA function is required at the organismal level. In other words, studies using miRNA mutants are the most reliable tool for assessing the biological function of miRNAs [13,14]. Null miRNA mutants are the gold standard for LOF studies. However, due to the very short sequences of miRNAs, most classic mutagenesis approaches are rendered ineffective for generating null miRNA mutants. In addition, there are many inherent challenges in performing experiments using whole organisms [105,114]. To date, only three organisms have a collection of miRNA mutants: *C. elegans*, and *Mus musculus*, and the model insect, *D. melanogaster* [105].

## 4. Use of *Drosophila* for Insect-Virus Interaction Studies

The genetically tractable *Drosophila melanogaster* is a well-established model organism. It is easy to maintain, has a short generation time and, with each female able to produce thousands of eggs in its lifetime [115,116], has numerous offspring [117,118], thus conferring relative ease in scaling up experiments for statistical power. Apart from the logistical advantages of using *D. melanogaster*, the use of this model organism has many technical advantages. This includes *D. melanogaster’*s compact genome which removes the complications presented by redundant genes and simplifies functional analyses [118]. Additionally, there is a readily available suite of *Drosophila* tools [119] including genetic tools for manipulation of the fruit fly genes such as: RNA interference (RNAi) for knockdown of target transcripts and conditional drivers such as Gal4/UAS system to express genes of interest [120]. Importantly, large collections of *Drosophila* stocks with a range of phenotypes and mutations are available in several publicly accessible repositories, thus permitting genotype to phenotype and molecular to organismal level studies. Bloomington Drosophila Stock Centre alone holds over 20,000 *Drosophila* lines. Of these stocks, more than 12,000 have insertion mutants, representing the majority of *Drosophila* genes [119,121].

Our knowledge on many aspects of insect biology is scaffolded on *D. melanogaster* studies. This is made possible by the high degree of conservation of *D. melanogaster* genes with the genes of the other insects, including mosquitoes. In addition, *D. melanogaster* exhibits genetic pathways and life cycles similar to mosquitoes [18,61,122,123]. Although *D. melanogaster* is not a vector for arboviruses, it is used as an experimental host to many medically and agriculturally important viruses including Rift Valley Fever Virus (RFV), Sindbis virus (SINV), DENV, WNV, and BTV [18,124,125,126,127,128,129,130,131]. As such, fruit flies have been vital in our understanding of the insect antiviral responses [18,132]. In particular, studies on *Drosophila* have contributed to our understanding of the involvement of the small interfering RNA (siRNA) pathway as a critical antiviral response in mosquitoes [18,132,133,134].

There are *Drosophila* stock collections with miRNA GOF and LOF available. Of which, there are lines generated using Gal4-UAS system for conditional expression of 165-180 miRNAs [135,136]; lines of *Drosophila* with overexpressed transgenes encoding multiple copies of miRNA target sites that sequester a total of 141 high-confidence miRNAs [137]; and a collection of *D. melanogaster* miRNA knockout mutants covering 130 individual miRNAs generated [138]. 

GOF and LOF miRNA lines enable a novel route for identification of miRNAs that are key players in insect-virus interaction (Figure 1, left panel). Instead of starting with miRNA profiling, the search for relevant miRNAs can begin with infection of fly lines and determining which infected fly lines exhibit marked changes in parameters of host-virus interaction, particularly host mortality and/or virus accumulation. The reminder that most miRNAs are modulators and fine-tuners rather than on-off switches should however be raised, and therefore only those miRNAs which are main regulators will likely exhibit drastic phenotypic changes.

The utility of the *Drosophila* miRNA mutants for the study of the host-virus interaction was recently demonstrated. In flies, miR-956-3p was identified as highly abundant with reduced abundance in the presence of the *D. melanogaster* natural pathogen Drosophila C virus. Using a LOF miR-956 mutant confirmed the functional impact of miR-956 down-regulation as reducing viral accumulation and delaying virus-induced mortality. Following bioinformatics analysis Ectoderm-expressed 4 (*Ect-4*) was identified as a target of miR-956 and functional analysis using *Ec-t4* LOF flies confirmed that it was involved in regulating the host-virus interaction [84]. The initial success herewith in using miRNA KO for uncovering miR-956 function in DCV pathogenesis in *D. melanogaster* is a proof of concept for using miRNA KO flies for determining endogenous host-virus interaction function of miRNA genes. Additionally, it shows that given the already available *Drosophila* tools, genotype to phenotype and molecular to organismal level miRNA studies are now possible. Interestingly, miR-956-5p has been found to be highly abundant in *Ae. albopictus* midgut [68] and the orthologues of Ect4 are found even in species that appeared some 500 million years ago [139] helps advance the case of *Drosophila* utility for miRNA functional studies. With around 80% of *Aedes* miRNA homologous with *Drosophila* miRNA and with 98% of insects sharing a conserved microRNA toolkit of 65 families exhibiting very low variation [140], it is possible that some of the miRNA-virus impacts observed in *Drosophila* may extend to other host-virus systems.

There are some limitations of the *Drosophila*-arbovirus infection model. For example, arbovirus infection of mosquitoes generally occurs through blood feeding, whereas experimental infection of *Drosophila* with arboviruses is usually achieved via injection and using higher doses of virus. In addition, because of the differences in the experimental *Drosophila* model and the natural arbovirus infection cycle it is clear that not all miRNA impacts on the host-virus interaction will be captured by the experimental model. However, the demonstrated similarities in the inducible immune responses between *Drosophila* and mosquitoes suggests that there may be overlap in the role of miRNAs as gene regulators that impact virus infection.

## 5. Conclusions

To date, studies focused the role of miRNAs is arbovirus/phytoarbovirus infection of non-insect hosts far exceeds amount of studies on infection of insect hosts. Given that understanding the role of miRNAs and their targets as modulators of the insect-arbovirus/phytoarbovirus interaction can open avenues for using miRNAs, their targets, and/or miRNA modulation pathways as novel approaches for managing arbovirus and phytoarbovirus infections, as well as for disrupting the epidemiological cycle of transmission, there is impetus for attention to be focused on insect hosts as well. Important information on miRNAs which are possibly involved in insect-virus interaction already exists even with just the few, and fragmentary, miRNA profiling studies carried out on arbovirus/phytoarbovirus infection of insects. Functional analysis of the differentially expressed miRNAs will make more meaningful use of the available data. While the use of the pertinent vector insects and their viruses remain the ultimate goal, and while there are physiological differences between *Drosophila* and other insects, the use of *Drosophila* may augment the studies on the role of miRNA in insect-arbovirus/phytoarbovirus interaction.

## Figures and Tables

**Table 1:** Summary of miRNA profile change studies in insect-arbovirus interaction.

	*Ae. aegypti*			*Ae. albopictus*			*Culex*		
	Whole Insect	Insect Parts	Cell	Whole Insect	Insect Parts	Cell	Whole Insect	Insect Parts	Cells
BTV						0.5 dpi [73]			
CHIKV		saliva, 10 dpi * [66]	1 dpi [74]		saliva, 10 dpi * [66]				
DENV	2, 4, 9 dpi [64]		3 dpi [75]	7 dpi * [65]	midgut, 1 dpi [68]				
WNV							14 dpi [76]		
ZIKV	2, 7, 14 dpi [67]								

* Denotes intra-thoracic inoculation of virus. Where not specified, whole mosquitoes are infected by bloodmeal.

**Table 2:** NGS-quantified differential abundance of *Aedes* miRNAs during virus infection.

	*Aae*-ZIKV	*Aae*-DENV	*Aal*-DENV	*Aal*-BTV	*Aal*-CHIKV	*Aal*-DENV *	*Aae*-CHIKV ^	*Aal*-CHIKV ^			*Aae*-ZIKV	*Aae*-DENV	*Aal*-DENV	*Aal*-BTV	*Aal*-CHIKV	*Aal*-DENV *	*Aae*-CHIKV ^	*Aal*-CHIKV ^
bantam/bantam-3p		↓	↓				↑	↓		miR-1			↓					
miR-263a/miR-263a-5p	↓		↓				↑	↑		miR-1-5p	↑							
miR-34/miR-34-5p			↑	↓			↑	↓		miR-1174							↓	
let-7		↓	↓					↑		miR-1175-3p		↓						
miR-1000/miR-1000-5p		↓			↓		↑			miR-12							↑	
miR-275/miR-275-3p			↓				↑	↑		miR-124-5p		↓						
miR-276/miR-276-3p			↓			↑	↑			miR-13							↑	
miR-281/miR-281-5p			↓				↓	↑		miR-15b						↑		
miR-2941	↓		↓				↓			miR-1767						↑		
miR-305/miR-305-5p	↓	↓					↑			miR-1889							↑	
miR-315					↓		↑	↑		miR-190							↑	
miR-317		↓	↓				↑			miR-193-5p						↑		
miR-8/miR-8-3p		↓	↓					↓		miR-252								↑
miR-927			↓				↑	↑		miR-275-5p					↑			
miR-957			↓				↑	↑		miR-276-1								↑
miR-989	↑		↓				↓			miR-277-5p			↓					
miR-999		↓					↑	↑		miR-279							↑	
miR-308/miR-308-5p	↓		↑				↑			miR-281a-3p		↓						
miR-10							↑	↑		miR-281a-5p		↓						
miR-100							↑	↑		miR-281b-3p		↓						
miR-125							↑	↑		miR-281c-5p		↓						
miR-14							↑	↑		miR-283							↓	
miR-184			↓				↓			miR-285							↑	
miR-1889-5p			↓	↓						miR-286a	↓							
miR-1890		↓					↑			miR-286b	↓							
miR-210		↓					↑			miR-2944b-5p	↓							
miR-263b/miR-263b-5p			↓				↑			miR-2a							↑	
miR-276-5p		↓	↑							miR-2c							↑	
mir-277							↑	↑		miR-2c-3p					↓			
miR-281-2-5p							↓	↑		miR-307							↑	
miR-2940/miR-2940-5p			↓				↓			miR-309a	↓							
miR-2940-3p	↑		↓							miR-33-5p		↓						
miR-2944b-3p	↓				↓					miR-3368-5p		↓						
miR-2945-3p		↓	↑							miR-34-3p		↑						
miR-2946	↓						↓			miR-3722-5p		↓						
miR-2b		↓					↑			miR-3811e-5p						↑		
miR-306/miR-306-5p			↓				↑			miR-4275-5p		↓						
miR-308-3p	↓	↓								miR-4448						↓		
miR-375	↓						↓			miR-4728-5p						↑		
miR-71/miR-71-5p	↑						↑			miR-5108-5p		↓						
miR-980/miR-980-3p	↓						↑			miR-5119-5p		↑						
miR-9a					↑		↑			miR-6134						↑		
miR-9c-5p		↓	↓							miR-622						↑		
miR-133							↑	↑		miR-79c-3p		↓						
miR-1891							↑	↑		miR-8-5p			↓					
mir-92b							↑	↑		miR-87-5p		↑						
										miR-927a					↑			
										miR-92a							↑	
										miR-932							↑	
										miR-932-5p		↓						
										miR-970							↑	
										miR-988-5p		↑						
										miR-993			↑					
										miR-996							↑	
										miR-998							↑	
										miR-9b			↓					
										miR-iab		↓						

*Aae*: *Ae. aegypti*; *Aal*: *Ae. albopictus*; *midgut sample; ^ saliva sample; *Ae. aegypti*-ZIKV, based on Table 1 [67]; *Ae. aegypti*-DENV, based on Table 1, applied 1.5-fold cut-off [64]; *Ae. albopictus*-DENV, based on Table 3 [65]; *Ae. albopictus*-BTV, based on Table 4, applied 100 TPM cut-off [73]; *Ae. albopictus*-CHIKV, based on Figure 1, applied 1.5-fold cut-off [74]; *Ae. albopictus*-DENV midgut, based on Table 5, applied 100 TPM cut-off [68]; *Ae. aegypti*-CHIKV saliva, based on Table 1, applied 1.5-fold and 100 TPM cut-off [66]; *Ae. albopictus*-CHIKV saliva, based on Table 1, applied 1.5-fold and 100 TPM cut-off [66].

**Table 3:** Functional analyses of miRNAs which are differentially abundant upon virus infection. All miRNA regulation assays are tested *in vivo* and all virus accumulation assays are tested ex vivo unless otherwise specified.

virus	miRNA	miRNA Regulation during Viral Infection		virus Accumulation upon Mimic Treatment					virus Accumulation upon Inhibitor Treatment					Target/Function
		Ae. aegypti	Ae. albopictus	1 dpi	2 dpi	3 dpi	4 dpi	5 dpi	1 dpi	2 dpi	3 dpi	4 dpi	5 dpi	
CHIKV	miR-12[66]	↑^s^10 dpi	≈ ^s^ 10 dpi						↑^#^	↓ ^#^				targets MCM6 & MCT1 [107]
	miR-125[66]	↑ ^s^10 dpi	↑ ^s^10 dpi						↑ ^#^	↓ ^#^	↑ ^#^			
	miR-184[66]	↓ ^s^10 dpi	≈ ^s^ 10 dpi						↓	↓ ^#^				
	miR-375[66]	↓ ^s^10 dpi	⊗^s^ 10 dpi							↓ ^#^				enhances DENV infection [108], up-regulates cactus, down-regulates REL1 [108]
	miR-2940[66]	≈ ^s^10 dpi	≈ ^s^10 dpi						↑ ^#^	↓ ^#^				up-regulates metalloprotease m41 ftsh [95], arginine methyltransferase [96], & DNA methyltransferase [97]
WNV	miR-2940-5p[98]		↓3 dpi & 5 dpi		↑	↑	↑	↑			↓		↓	
DENV	miR-252[109]		↑1 dpi & 3 dpi	↓		↓			↑		↑			targets DENV-2 E protein [109]
	miR-281[94]		↑ 4 dpi↑^wm & m^ 4 dpi & 7 dpi		↑					↓		↓ *		targets 5′-UTR of DENV2 to enhance viral replication [94]
	miR-4728^m^[68]		↑ ^wm^1 dpi			↑	↑ **				↓			

↑ up-regulation; ↓ down-regulation; ≈: less than 1.5-fold change; ⊗: not detected; Experiments done in C6/36 except when otherwise indicated. ^s^ saliva sample; ^m^ midgut sample; ^wm^ whole mosquito sample; **^#^** AAG-2 cell used; * in vivo (4 dpi and also 7 dpi); ** increase in cytopathic activity noted.
